# Doxorubicin hydrochloride enhanced antitumour effect of CEA‐regulated oncolytic virotherapy in live cancer cells and a mouse model

**DOI:** 10.1111/jcmm.15966

**Published:** 2020-10-14

**Authors:** Boduan Xiao, Chang Ying, Yongyi Chen, Fang Huang, Binrong Wang, Huiling Fang, Wan Guo, Tao Liu, Xiumei Zhou, Biao Huang, Xinyuan Liu, Yigang Wang

**Affiliations:** ^1^ Xinyuan Institute of Medicine and Biotechnology School of Life Sciences and Medicine Zhejiang Sci‐Tech University Hangzhou China; ^2^ Institute of cancer research and basic medical sciences of Chinese Academy of Sciences Cancer hospital of University of Chinese Academy of Sciences Zhejiang cancer hospital Hangzhou China; ^3^ Department of Pathology Zhejiang Provincial People’s Hospital Hangzhou China; ^4^ Department of Otolaryngology Guangdong General Hospital Guangdong Academy of Medical Sciences Guangzhou China

**Keywords:** CD55‐TMn, CEA, doxorubicin hydrochloride, liver cancer, oncolytic virotherapy

## Abstract

Oncolytic adenovirus (OA) has attracted increasing attention due to their specific proliferation in tumour cells and resulting in lysis of tumour cells. To further improve the antitumour effect of OA, in this study, we combined CD55‐TRAIL‐IETD‐MnSOD (CD55‐TMn), a CEA‐controlled OA constructed previously, and chemotherapy to investigate their synergistic effect and possible mechanisms. MTT assay was performed to detect antitumour effects. Hoechst 33 342 and flow cytometric analysis were used to examine cell apoptosis. Western blotting was performed to examine cell pyroptosis and apoptosis mechanism. Animal experiment was used to detect antitumour effect of doxorubicin hydrochloride (Dox) combined with CD55‐TMn in vivo. We firstly found that Dox promotes gene expression mediated by CEA‐regulated OA and virus progeny replication by activating phosphorylation of Smad3, and Dox can enhance antitumour effect of CEA‐regulated CD55‐TMn by promoting cell apotopsis and cell pyroptosis. Thus, our results provide an experimental and theoretical basis on tumour therapy by combination treatment of the oncolytic virotherapy and chemotherapy and it is expected to become a novel strategy for liver cancer therapy.

## 1 INTRODUCTION

Recently, oncolytic virus has widely been studied throughout the world, such as oncolytic adenovirus (OA), herpes simplex virus and poxvirus that can specifically proliferate in tumour cells and finally lead to lysis of the tumour cells.^1^ To enhance the antitumour effect of the oncolytic virus, cancer‐targeting gene virotherapy (CTGVT) as a novel strategy has been proposed by our group, which is constructed by inserting an antitumour gene into an oncolytic viral vector (OA).^2^, ^3^ Besides, we have found that oncolytic viral vector carrying two different genes has stronger antitumour effect than oncolytic viral vector carrying any single gene, such as ZD55‐TRAIL‐IETD‐Smac and CD55‐TRAIL‐IETD‐MnSOD (CD55‐TMn).^4^, ^5^ Tumour necrosis factor‐related apoptosis‐inducing ligand (TRAIL) is considered as a potential effective antitumour agent by induction of tumour cell apoptosis.^6^, ^7^ Previous researches have reported that OV‐mediated TRAIL has a good antitumour effect in hepatocellular carcinoma (HCC),^8^ colorectal cancer^9^ and lung adenocarcinoma.^10^ Manganese superoxide dismutase (MnSOD) is a number of metal superoxide dismutase family. Our previous study showed complete suppression of tumour xenograft by combined OA‐mediated MnSOD with TRAIL gene virotherapy via promoting tumour cell apoptosis.^11^ Some reports suggest that MnSOD effectively inhibits tumour growth in HCC,^12^ colorectal cancer^13^ and pancreatic cancer.^14^

Doxorubicin hydrochloride (Dox) is a kind of traditional chemotherapy drugs, and it has wide antitumour spectrum, which is used in a variety of cancer chemotherapy.^15^ Dox belongs to the cycle non‐specific drug that can suppress cell cycle of tumour cells. However, high‐dose Dox often causes cardiac toxicity.^16^ Thus, how to improve the Dox sensitivity for chemotherapy and reduce its dose to achieve the best antitumour effect is the problem to be solved.

Previously, our study has showed that CD55‐TMn has a strong antitumour effect in mouse tumour xenograft.^5^ In this study, we further explored how to enhance the antitumour effect of CD55‐TMn. Thus, we found Dox could enhance antitumour effect of CD55‐TMn in vitro and in vivo by a synergistic approach. Our results provide an experimental and theoretical basis on tumour therapy by combination treatment of the oncolytic virotherapy and chemotherapy.

## 2 MATERIALS AND METHODS

### 2.1 Cells and culture

The HEK293 (containing the E1A region of) was acquired from Microbix Biosystems Inc (Toronto, Ontario, Canada). Human HCC cell lines Hep G2, Hep 3B and PLC/PRF/5 and human normal liver cell line L‐02 were purchased from the Shanghai Cell Collection (shanghai, China). The HEK293 and L‐02 were cultured in Dulbecco's modified Eagle's medium (DMEM; GIBCO BRL, Grand Island, NY) supplemented with 10% heat‐inactivated foetal bovine serum (FBS; GIBCO BRL). The Hep G2, Hep 3B and PLC/PRF/5 were cultured in DMEM supplemented with 5% FBS. All cell lines were cultured at 37°C in a 5% CO2 humidified incubator.

### 2.2 Cell viability assay

The 5000 cells were inoculated into 96‐well plates, and after cultured 12 hours, they were treated with CD55‐TMn, Dox (Beyotime, Nantong, China), or a combination of CD55‐TMn and Dox. All cells were incubated at 37°C in a 5% CO2. At the indicated time after treatment, 15 µl solution containing 3‐(4,5‐dimethylthiazol‐2‐yl)‐2,5‐diphenyltetrazolium bromide (MTT, 0.5 mg/ml) was added to each well. After 4h in the incubator, the cell supernatants were completely removed and each well was added with 150 µl dimethyl sulphoxide. After mixing thoroughly, the 96‐well plates were read by a microplate reader with absorbance for 490 nm (TECAN, Austria).

### 2.3 Apoptotic cell staining

The 5 × 10^5^ cells were inoculated into 6‐well plates, and after one night, they were treated with CD55‐TMn, Dox, or combination of CD55‐TMn and Dox. All cells were incubated at 37°C in a 5% CO2. After 48 hours, the cells were incubated with Hoechst 33 342 (Beyotime, Nantong, China) for 10min and then washed twice with phosphate buffered saline (PBS). Subsequently, the cells were observed under a fluorescence microscope.

### 2.4 Western blot analysis

The 5 × 10^5^ cells were inoculated into 6‐well plates, and after one night, they were treated with CD55‐TMn, Dox, or combination of CD55‐TMn and Dox. All cells were incubated at 37°C in a 5% CO2. After 48 hours, all cells were cracked and proteins were collected. Concentration of protein collections was determined by Pierce BCA protein assay kit (Thermo Fisher Scientific, Waltham, MA, USA). Subsequently, protein is separated by SDS‐polyacrylamide gel electrophoresis and transferred to polyvinylidene difluoride membranes. The membranes were blocked with 5% non‐fat dry milk and incubated with primary antibodies. After one night, the membranes were incubated with secondary antibodies. Then, expression of proteins was detected by Odyssey infrared imaging system (LI‐COR Biosciences Inc, Lincoln, NE, USA). GAPDH, Smad3, caspase‐8, caspase‐3, caspase‐9, poly‐ADP‐ribose polymerase (PARP), TRAIL, cleaved caspase‐9, cleaved caspase‐3, TNF‐α, IL‐1β and XIAP antibodies were purchased from Cell Signaling Technology (Danvers, MA, USA). E1A and MnSOD antibodies were obtained from Santa Cruz Biotechnology (Santa Cruz, CA, USA). CEA antibodies were obtained from Affinity Biologicals Inc (Ancaster, Canada). GSDME, GSDME‐N and P‐Smad3 antibodies were obtained from Abcam plc (Cambridge, UK).

### 2.5 Flow cytometric analysis

The 5 × 10^5^ cells were inoculated into 6‐well plates, and after one night, they were treated with CD55‐TMn, Dox, or combination of CD55‐TMn and Dox. All cells were incubated at 37°C in a 5% CO2. After 48 hours, the cells were trypsinized and harvested. Aliquots of cell were resuspended with 500 µl binding buffer and stained with Annexin V FITC/PI (BD Biosciences, San Jose, CA, USA) based on the manufacturer's instructions. The cells were detected immediately by a fluorescence‐activated cell sorting (BD Biosciences) assay.

### 2.6 Animal experiment

Animal experiments were performed on the basis of the guide for regulations and standards of Experiment Animal of the US Department of Agriculture and National Institutes of health. The female BALB/c nude mice about 4 weeks old were purchased from the Shanghai Experimental Animal Center (Shanghai, China). To establish the tumour xenograft model, PLC/PRF/5 cells were injected subcutaneously into the right flank of nude mice. When the tumour arrived at 80‐120 mm^3^, the nude mice were divided randomly into four groups. Subsequently, the nude mice were injected with PBS, Dox (1.2 mg/kg), CD55‐TMn (5 × 10^8^ pfu) and CD55‐TMn combined with Dox. CD55‐TMn (5 × 10^8^ pfu) was injected into mouse via an intratumoural manner for two times once a day for two consecutive days. Subsequently, the Dox was injected every five days for a total of three injections. After injecting virus, the tumour size was measured with a vernier calliper every five days.

### 2.7 Histopathology, IHC and TUNEL assay

For analysis of histopathology, mice have been randomly selected from each group and were killed after 20 days of the first treatment in different groups. Tumour tissue, heart, liver, kidney and spleen were harvested and fixed in 5% paraformaldehyde, dehydrated with gradient increasing ethanol concentrations and embedded in paraffin wax, which were cut in 5‐µm sections. The sections were stained with haematoxylin and eosin for histological analysis. The sections were incubated with anti‐TRAIL antibodies and then incubated with the avidin‐biotin‐peroxidase complex reagent (Vector Laboratories, Burlingame, CA, USA) for the IHC analysis. Haematoxylin was used as a counterstain. In situ apoptosis detection kit (Sino‐American Biotechnology Co., Luoyang, China) was used to stain apoptotic cell tumour tissue sections on the base of the manufacture's instruction for the TUNEL assay. All sections were counterstained with haematoxylin.

### 2.8 Statistical analysis

All experimental data were appeared as mean ± SD The differences were assessed by analysis of variance and Student's t test, and the data were considered statistically significant at *P* < 0.05.

## 3 RESULT

### 3.1 Synergistic cytotoxic effect of combination of CD55‐TMn and Dox in HCC cells

**FIGURE 1:**
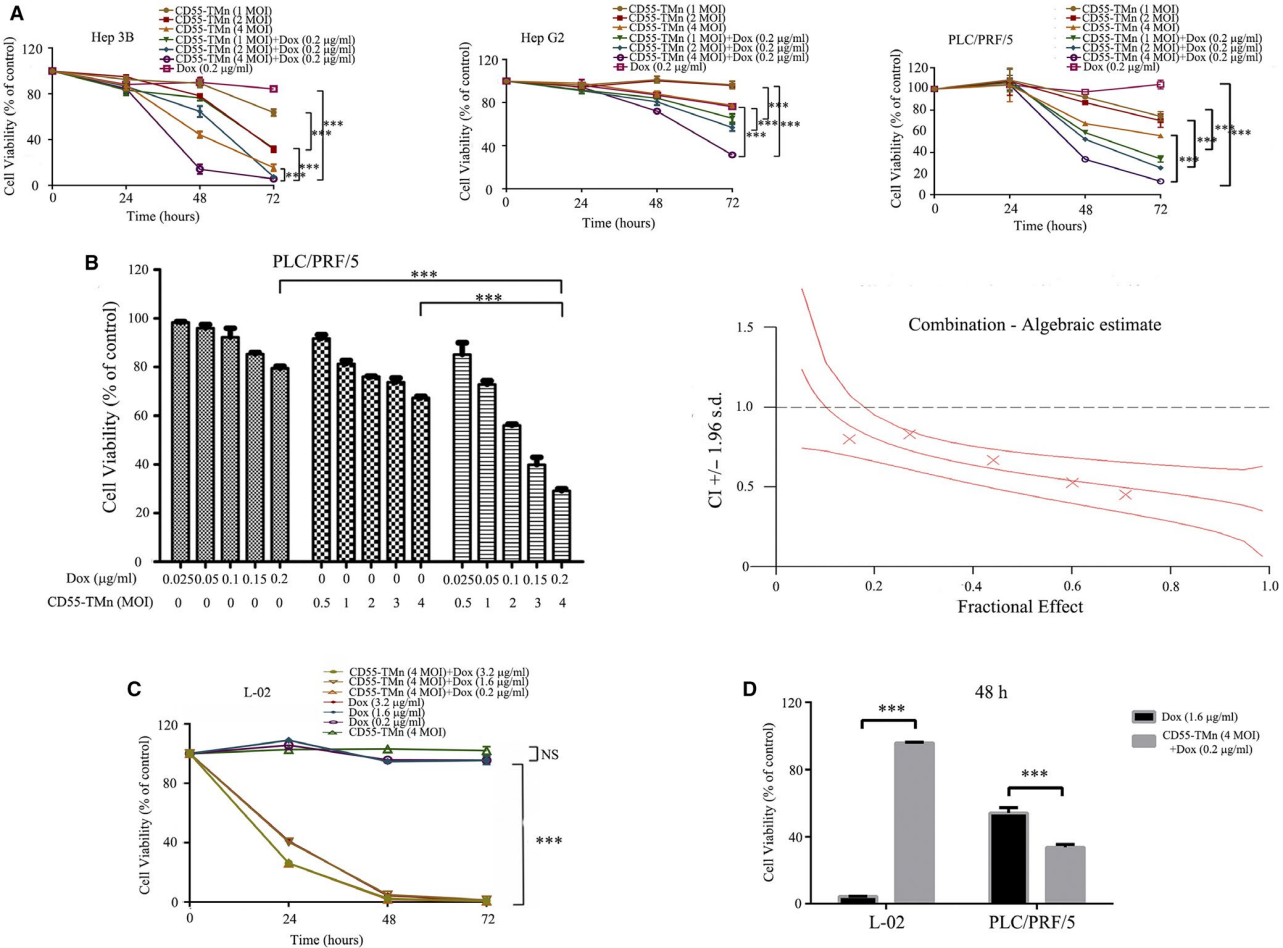
Synergistic cytotoxic effect of the combination of CD55‐TMn and Dox in HCC cells. A, Cell viability of Hep 3B, Hep G2 and PLC/PRF/5 cell was examined via an MTT assay. B, The quantitated data of combination treatment were processed using CalcuSyn software, and the CI values of the treatment groups are represented by X‐marks. C, Cell viability of L‐02 cell was examined via an MTT assay. D, Cell viability of L‐02 cell and PLC/PRF/5 for 48 h was examined via an MTT assay. All data are presented as the mean ± standard deviation. n =3. **P < 0.01; ***P < 0.001. NS, not significant

We detected the cytotoxicity and synergistic effect of the combination of CD55‐TMn and Dox in HCC cell line Hep G2, Hep 3B and PLC/PRF/5 treated with CD55‐TMn at various MOIs (1, 2 and 4) and Dox (0.2 µg/ml). The result showed that the combination of CD55‐TMn and Dox had greater inhibitory effect than CD55‐TMn alone and Dox alone (Figure 1A). The various HCC cell viability rate of combination treatment compared to any treatment alone was significantly decreased in a time‐dependent manner. Moreover, the antitumour effect of combination therapy with the CD55‐TMn and Dox was synergistic in PLC/PRF/5 cells (Figure 1B), which were evaluated by the CalcuSyn analysis. Thus, our data showed that oncolytic virotherapy works well in vitro.

To detect the safety and cytotoxicity of Dox in normal cells and HCC cells, respectively, we conducted cell viability assay by different treatments. The combination of Dox (0.2 µg/ml) and CD55‐TMn (4 MOI) or Dox (0.2 µg/ml) alone and CD55‐TMn (4 MOI) alone did not affect normal L‐02 cell growth with that the cell viability was > 95% after 72h, and there is no significant difference in toxic effects between high‐dose Dox (1.6 µg/ml or 3.2 µg/ml) combined CD55‐TMn (4 MOI) and high‐dose Dox (1.6 µg/ml or 3.2 µg/ml) in L‐02 cell (Figure 1C). In addition, we compared the combinational effect of Dox plus CD55‐TMn and medium‐dose Dox alone between normal cells and HCC cells for 48 h. The results showed that cell viability of L‐02 cells by the combination treatment of Dox (0.2 µg/ml) and CD55‐TMn (4 MOI) was up to 95% than that of 4.2% by with Dox (1.6 µg/ml) treatment (Figure 1D). In comparison, the combination of Dox (0.2 µg/ml) and CD55‐TMn (4 MOI) resulted in about 65% cell death in HCC PLC/PRF/5 cells, while Dox (1.6 µg/ml) alone only caused about 46% cytotoxicity effect (Figure 1D). These results suggest that oncolytic virotherapy is safer in vitro.

### 3.2 DOX enhanced virus progeny replication of CEA‐regulating virus through activating phosphorylation of Smad3

**FIGURE 2:**
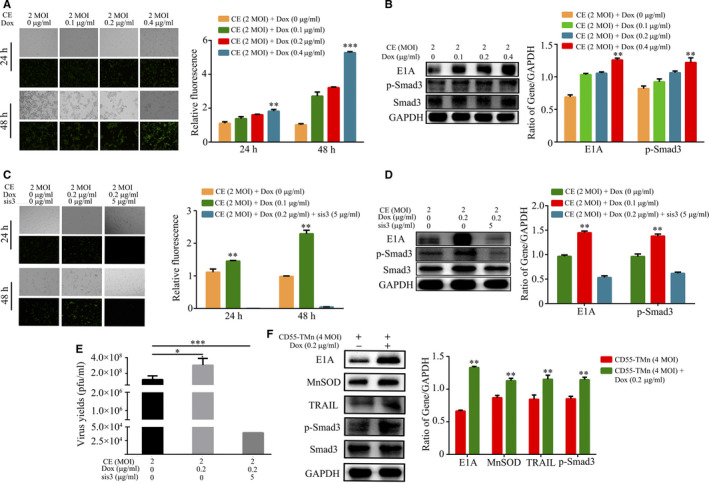
Dox enhances gene expression and virus progeny replication of CD55‐TMn through activating phosphorylation of Smad3. (A) and (C) The bright‐field images and the EGFP fluorescence images were, respectively, captured by using a light microscope and a fluorescence microscope (0.2 mm fields; magnification, x200). (B) and (D) The E1A, p‐Smad3 and Smad3 with difference dose Dox were detected by the Western blot analysis after 48 h. GAPDH was used as the internal control. E, The virus yields with the treatment of Dox alone or Dox and SIS3 were detected by TCID50 analysis after 48 h. F, The expression of E1A, MnSOD, TRAIL, p‐Smad3 and Smad3 with difference dose Dox was detected by the Western blot analysis after 48 h, GAPDH was used as the internal control. All data are presented as the mean ± standard deviation. n = 3. *P < 0.05, **P < 0.01, ***P < 0.001. All CE is CD55‐EGFP

Our research results first showed inhibition of TGF‐β receptor type I/II and phosphorylation of Smad3 suppresses proliferation of CEA‐regulated oncolytic adenovirus CD55 in HCC (Fig. S1). In addition, Dox promoted CEA expression and phosphorylation of Smad3 in HCC (Fig. S2). To further verify whether Dox promoted gene expression mediated by CEA‐regulated OA by promoting phosphorylation of Smad3, we used various dose Dox to treat CD55‐EGFP‐infected PLC/PRF/5 cells. The result showed that E1A expression, EGFP expression and phosphorylation of Smad3 were increased in PLC/PRF/5 cells infected by CD55‐EGFP in a dose‐dependent manner after Dox treatment (Figure 2A and B). Furthermore, we also confirmed whether Smad3 inhibition can affect Dox‐mediated gene expression of HCC cells infected by CEA‐regulated OA. As shown in Figure 2C and D, treatment of Smad3 inhibitor SIS3 significantly lowered E1A expression, Smad3 phosphorylation and the fluorescence intensity of EGFP expression in PLC/PRF/5 cells infected by CD55‐EGFP, which provides another evidence that inhibition of phosphorylation of Smad3 restrained gene expression mediated by CEA‐regulated OA via inhibition of CEA expression. In addition, analysis of virus progeny replication has indicated that Dox significantly enhanced replication of CD55‐EGFP, and addition of SIS3 inhibitor significantly restrained replication of CD55‐EGFP in PLC/PRF/5 cells (Figure 2E). Notably, Dox treatment also obviously increases expression of E1A, TRAIL and MnSOD and the phosphorylation of Smad3 in PLC/PRF/5 cells infected by CD55‐TMn (Figure 2F).Thus, the data suggest that Dox can enhance gene expression mediated by CEA‐regulated OA and virus progeny replication via activating phosphorylation of Smad3, indicating that Dox can improve the level of phosphorylation of Smad3 of hepatocarcinoma cells to make it more suitable for proliferation and exogenous gene expression of oncolytic adenovirus.

### 3.3 The combination of CD55‐TMn and DOX induced pyroptosis through caspase‐3 cleavage of GSDME

**FIGURE 3:**
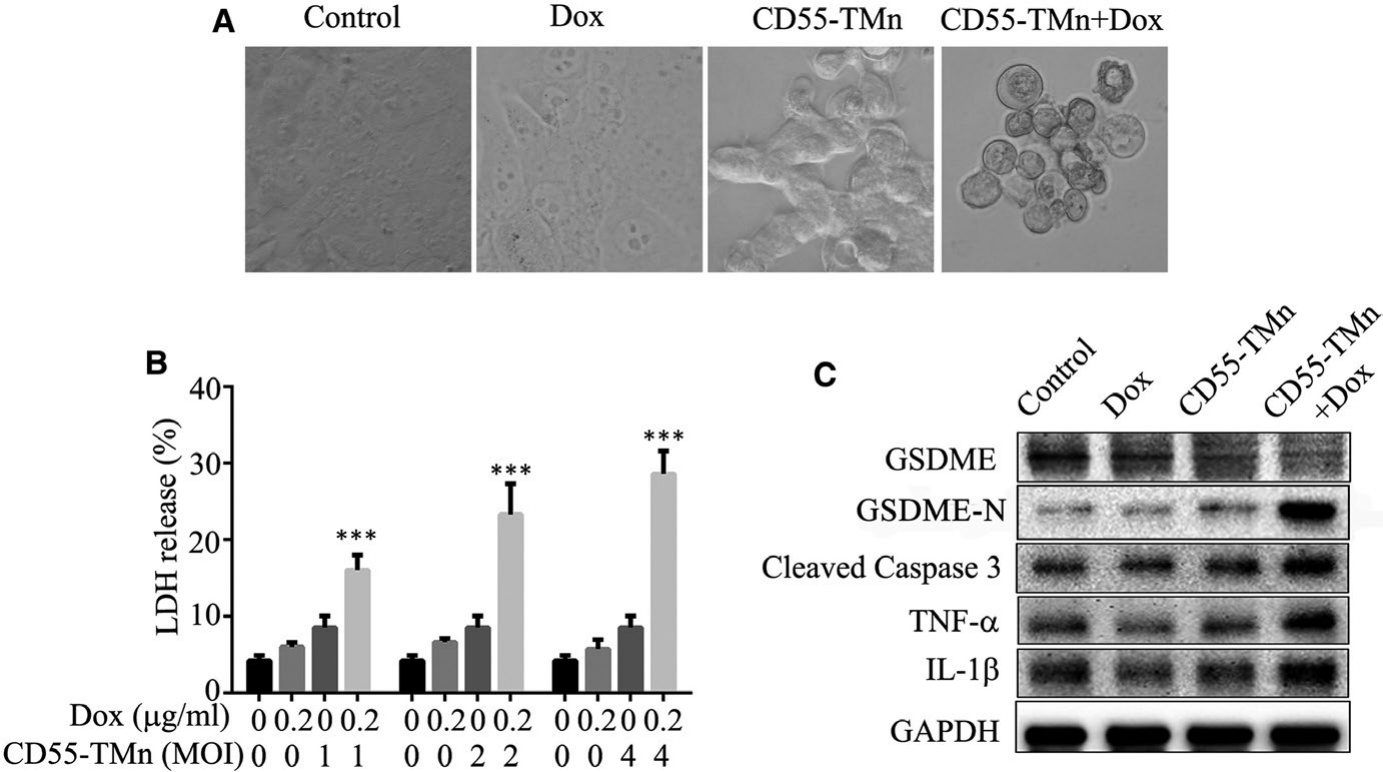
The combination of CD55‐TMn and Dox induced pyroptosis through caspase‐3 cleavage of GSDME. A, Phase‐contrast imaging assay of pyroptosis. B, LDH release‐based cell death. C, Caspase‐3 cleavage of GSDME was detected by Western blotting, and GAPDH was used as the internal control. All data are presented as the mean ± standard deviation. n = 3. *P < 0.05, **P < 0.01, ***P < 0.001

Pyroptosis is a form of cell death that is critical for immunity. In this study, the PLC/PRF/5 cells treated with the combination of CD55‐TMn and Dox showed evident swelling with characteristic large bubbles from the plasma membrane (Figure 3A). In addition, LDH assay showed the combination of CD55‐TMn and Dox induced more releases of lactate dehydrogenase (LDH) compared to Dox alone or CD55‐TMn alone (Figure 3B). To verify that the combination of CD55‐TMn and Dox induced pyroptosis through caspase‐3 cleavage of GSDME, the Western blotting was performed. The results showed cleaved caspase‐3, GSDME‐N, TNF‐α and IL‐1β were increased and GSDME were decreased in the combination of CD55‐TMn and Dox compared to Dox or CD55‐TMn (Figure 3C), suggesting the combination of CD55‐TMn and Dox induced pyroptosis through caspase‐3 cleavage of GSDME.

### 3.4 DOX significantly enhanced cell apoptosis in HCC infected with CD55‐TMn

**FIGURE 4:**
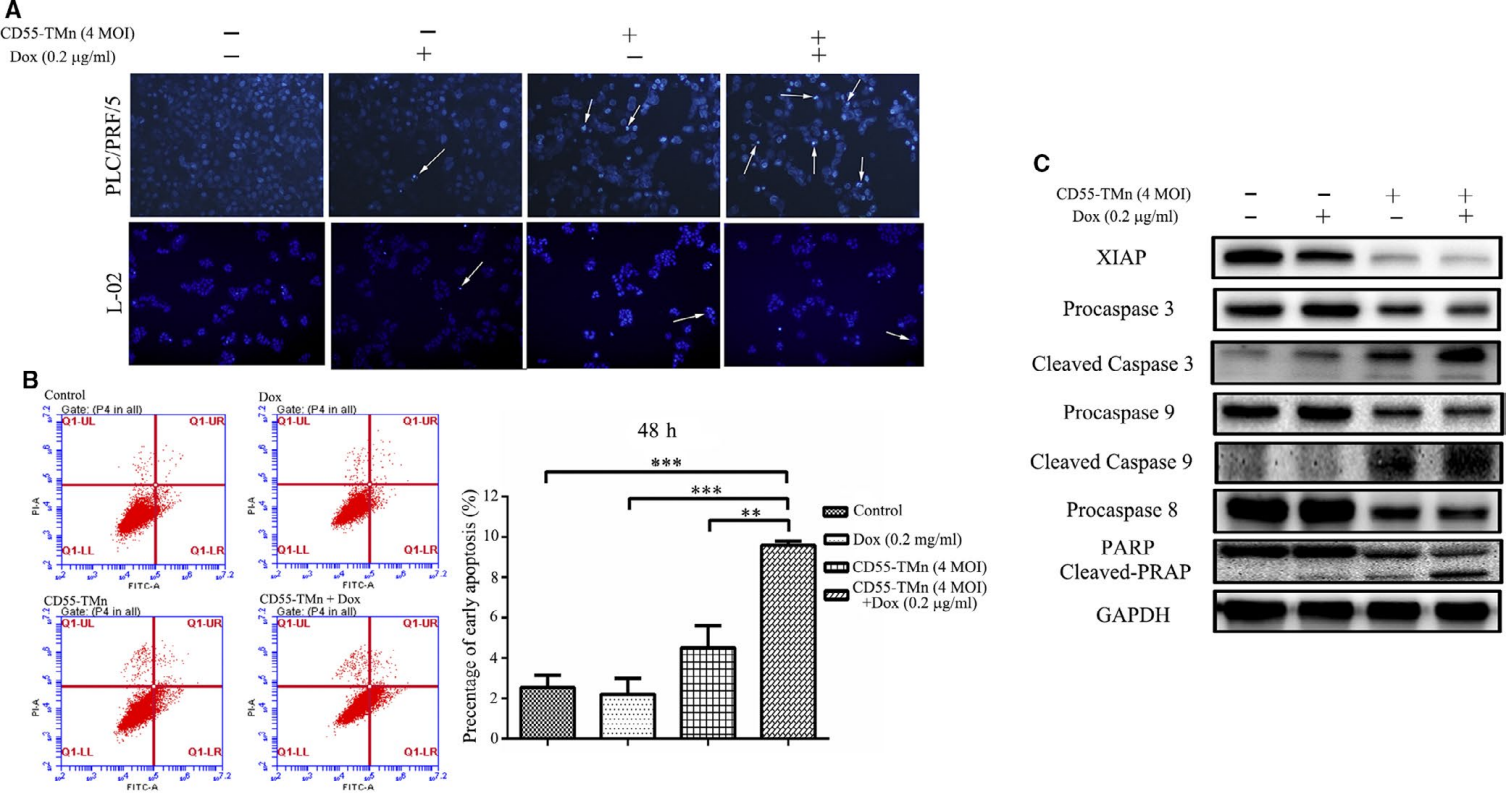
Dox significantly enhanced cell apoptosis in HCC infected with CD55‐TMn (A) After 48 h, nuclear fragmentation (arrows) was observed in PLC/PRF/5 cells (0.2 mm fields; magnification, x200) and L‐02 (0.2 mm fields; magnification, x100) cells in different treatment groups using Hoechst staining under an inverted fluorescence microscope. B, The percentage of apoptotic cells was detected using flow cytometric analysis. The data are presented as the mean ± standard deviation. n = 3. *P < 0.05, **P < 0.01, ***P < 0.001. C, Apoptosis‐associated protein expression was detected by Western blotting. GAPDH was used as the internal control

To further confirm whether Dox can promote cell apoptosis induced by CEA‐regulated OA, we conducted cell apoptotic analysis. First, Hoechst 33 342 staining was utilized to detect apoptotic morphological changes in the PLC/PRF/5 cells. The result showed that compared to CD55‐TMn or Dox alone, combination treatment of CD55‐TMn and Dox can emerge more nuclear fragmentation, formation of apoptotic body, chromatin condensation, and induce more cell apoptosis (Figure 4A). Remarkably, normal liver L‐02 cells with the treatment of CD55‐TMn, Dox or the combination therapy displayed the few signs of apoptosis (Figure 4A). The flow cytometry was further used to detect apoptosis in the PLC/PRF/5 cells, indicating that compared to 2.1% early cell apoptosis rate of Dox or 4.5% early cell apoptosis rate of CD55‐TMn, the combination treatment of CD55‐TMn and Dox can significantly induce more cell apoptosis and the early cell apoptosis rate was up to 9.6% (Figure 4B).

To explore the underlying mechanism in cell apoptosis, caspase signalling pathway was examined by Western blot analysis in the PLC/PRF/5 cells after 48 hours of various treatments. The result showed that CD55‐TMn induced obvious activation of caspase signalling pathway, and Dox treatment largely enhanced the cleavage of procaspase‐9, procaspase‐8, procaspase‐3, XIAP and PARP in PLC/PRF/5 cells infected by CD55‐TMn, revealing that the combination treatment can more effectively activate the caspase signalling pathway to induce the cell apoptosis that can enhance their antitumour effects (Figure 4C). Therefore, Dox can enhance apoptosis inducement effect of CD55‐TMn, and the combination treatment can more effectively kill the tumour cells via inducing cell apoptosis compared with each treatment alone.

### 3.5 Completed inhibition of HCC xenograft in mouse model by the combination of CD55‐TMn and DOX

**FIGURE 5:**
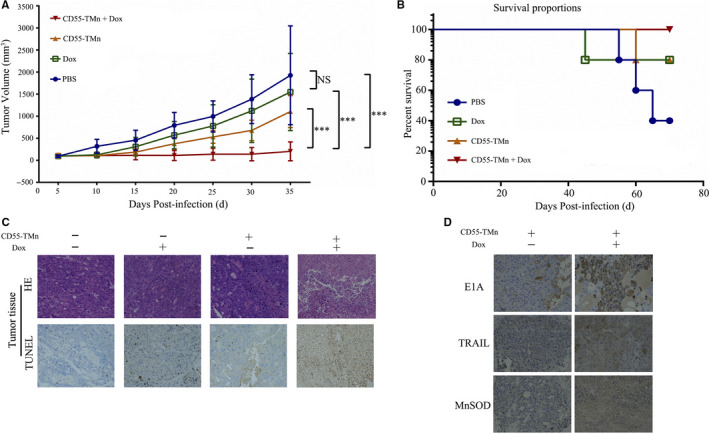
Oncolytic virotherapy model demonstrates excellent antitumour effects in vivo. A, The tumour volume was measured every 5 days using the formula V (mm3)=1/2 × length × width 2. Data are presented as the mean ± standard deviation, n = 5. *P < 0.05, **P < 0.01, ***P < 0.001. B, The mouse survival rate in different treatment groups. C, The cellular necrotic areas in the tumours were detected using HE staining, and the apoptosis of tumour sections was assayed using TUNEL staining. Magnification, x200. D, The expression of E1A, MnSOD and TRAIL in vivo was detected by the IHC analysis. Magnification, x200. E, The toxicity to heart, liver, kidney and spleen tissues was detected by HE staining. Magnification, x200

Dox can improve effective cytotoxic effect and apoptotic effect of CD55‐TMn in HCC cells. To further evaluate whether Dox can enhance antitumour efficacy of CD55‐TMn in vivo, PLC/PRF/5 cell xenograft model was established, and various treatments were performed. The result manifested that the tumour growth was more effectively inhibited in the combination treatment of CD55‐TMn and Dox, and tumour volume was only 201 mm^3^ compared with the 1926 mm^3^ for PBS, the 1546 mm^3^ for Dox and the 1104 mm^3^ for CD55‐TMn after treatment for 30 days (Figure 5A). Notably, there was no statistical difference in the tumour growth inhibition between the PBS and Dox group, implying that the dosage of Dox (1.2 mg/kg) alone almost did not suppress the tumour growth, but can greatly enhance the antitumour effects of CD55‐TMn in vivo. Moreover, all mice were alive in the combination treatment group and the survival rate was 100%, while the survival rate in PBS group, the Dox group and CD55‐TMn were 40, 80 and 80%, respectively, indicating that the combination treatment do not reduce mouse survival rate (Figure 5B). Further, we have performed the IHC and histology analysis on a single mouse from each treatment group. Compared to other treat‐group alone, Dox treatment increased cell apoptosis in tumour tissue treated with CD55‐TMn via TUNEL analysis, and the combination therapy can induce more severe cytopathic effects of tumour tissue that was examined by haematoxylin and eosin (HE) staining (Figure 5C). Moreover, the expression of E1A, TRAIL and MnSOD was increased in tumour mass with the treatment of Dox plus CD55‐TMn compared to CD55‐TMn alone in vivo (Figure 5D). In clinicopathologic analysis of normal organ tissue by HE staining, combination therapy almost did not cause the cytopathic effects in the liver, kidney, spleen and heart, implying that the addition of Dox had no effect in the normal tissue treated with CD55‐TMn and had a good security (Fig. S3).

## 4 DISCUSSION

Currently, oncolytic virotherapy becomes one of the most promising therapeutic strategies for solid malignancies. Up to date, there are more than 48 clinical trials for oncolytic virus alone or combination therapy in various solid tumours. In past several years, the combination of oncolytic virotherapy and cancer immunotherapy made a breakthrough progress in clinical trials. The commonest immunotherapeutic approach is use of immune checkpoint inhibitors (ICI), especially the combination of oncolytic virus T‐Vec and PD‐1 ICI pembrolizumab^17^ or CTLA‐4 ICI ipilimumab^18^ for advanced melanoma. Another study showed that oncolytic virus, as a neoadjuvant, sensitizes triple‐negative breast cancer before surgery to immune checkpoint therapy.^19^ To be noted, the common chemotherapy drug dimethyl fumarate can potentiate oncolytic virotherapy in xenograft tumour models through enhancing viral infection of cancer cell lines as well as human tumour biopsies,^20^ and a tumour targeting oncolytic adenovirus can improve therapeutic outcomes in chemotherapy resistant metastatic human breast carcinoma.^21^

In this study, we have found that the combination of Dox and CEA‐regulated OA CD55‐TMn exerted the synergic antitumour effect in HCC cells, compared to less than 10% inhibitory rate for Dox alone. Further, Dox promoted gene expression mediated by CEA‐regulated OA and virus progeny replication by activating phosphorylation of Smad3. Notably, IL‐1β expression is associated with TLR4, and the Dox combining CD55‐TMn promoted IL‐1β expression in HCC cell, suggesting the combination of CD55‐TMn and Dox has the ability to combine immunogenicity with oncolysis. The combination of CD55‐TMn and Dox exerted a synergistic effect and can more effectively inhibit growth of liver cancer via promoting cell pyroptosis and cell apoptosis. In the animal models, the tumour volume with treatment of Dox alone did not differ from the PBS group, but in the combination treatment of CD55‐TMn and Dox, the tumour volume was significantly smaller than any single treatment groups, showing statistically significant difference. Additionally, Dox has little cytotoxic effects on normal live cells and cell viability remained above 90%. The normal organ tissue, including liver, kidney, spleen and heart, did not cause obvious pathological phenomenon with the treatment of Dox, implying that it causes little toxic effect such as cardiotoxicity and displays better security. Thus, our data laid a foundation for the combinational treatment of chemotherapy Dox and CEA‐regulated CD55‐TMn in HCC.

Although great progress has been made in the field of oncolytic virotherapy, including cancer ‐targeting gene virotherapy strategy proposed by our group that was based on oncolytic adenovirus using the tumour‐specific promoters instead of E1A promoter to enhance the targeting ability and safety of adenoviruses,^5^, ^22^, ^23^, ^24^ there are some issues to restrain its application in cancer therapy. First, the heterogeneity of oncolytic adenoviruses may induce an inflammatory response. Adenovirus also triggers an anti‐viral effect of the immune system to reduce bioavailability. Moreover, neutralizing antibody to adenovirus existing in human bodies largely weakens oncolytic adenovirus‐mediated antitumour effect. These disadvantages of oncolytic adenoviruses need to be further resolved. Thus, the various combination therapies with oncolytic adenovirus were proposed, such as nanomaterial‐oncolytic adenovirus combination therapy,^25^, ^26^ immunocyto‐oncolytic virus combination therapy^27^, ^28^, ^29^ and chemotherapeutic drug‐oncolytic virus.^30^, ^31^, ^32^, ^33^ These combination strategies provide new ideas for future oncolytic virotherapy of tumours. Additionally, previous studies have focused on the identification of tumour biomarkers based on RNAs, which may provide the possibility of improving the antitumour effect of oncolytic virotherapy.^34^, ^35^, ^36^, ^37^, ^38^

In summary, we firstly found that Dox promotes gene expression mediated by CEA‐regulated OA and virus progeny replication by activating phosphorylation of Smad3, and Dox can enhance antitumour effect of CEA‐regulated CD55‐TMn by promoting cell apotopsis and cell pyroptosis. Thus, our results provide an experimental and theoretical basis on tumour therapy by combination treatment of the oncolytic virotherapy and chemotherapy, and it is expected to become a novel strategy for liver cancer therapy.

## CONFLICT OF INTEREST

We have no conflicts of interest to declare.

## AUTHORS’ CONTRIBUTION

Boduan Xiao: Conceptualization (equal); Data curation (equal); Formal analysis (equal); Investigation (equal); Methodology (equal); Software (equal); Writing‐original draft (equal). Chang Ying: Formal analysis (equal); Investigation (equal); Methodology (equal). Yongyi Chen: Methodology (equal); Software (equal). Fang Huang: Funding acquisition (equal); Methodology (equal); Resources (equal). Binrong Wang: Investigation (equal); Methodology (equal). Huiling Fang: Investigation (equal); Methodology (equal). Wan Guo: Investigation (equal). Tao Liu: Funding acquisition (equal); Methodology (equal). xiumei zhou: Visualization (equal). Biao Huang: Conceptualization (equal); Supervision (equal). Xin‐yuan Liu: Conceptualization (equal); Supervision (equal). Yigang Wang: Conceptualization (lead); Data curation (equal); Project administration (equal); Supervision (equal); Writing‐review & editing (equal).

## Supporting information

Fig S1

Fig S2

Fig S3

## References

[1] Acunzo M , Visone R , Romano G , et al. miR‐130a targets MET and induces TRAIL‐sensitivity in NSCLC by downregulating miR‐221 and 222. Oncogene. 2012;31:634‐642. 21706050 10.1038/onc.2011.260 PMC3719419

[2] Liu X . A new strategy for cancer therapy: Targeting gene‐virotherapy of cancer. Chinese Journal of Cancer. Biotherapy. 2001;8:1

[3] Zhang ZL , Zou WG , Luo CX , et al. An armed oncolytic adenovirus system, ZD55‐gene, demonstrating potent antitumoral efficacy. Cell Res. 2003;13:481‐489. 14728805 10.1038/sj.cr.7290191

[4] Wang SB , Tan Y , Lei W , et al. Complete eradication of xenograft hepatoma by oncolytic adenovirus ZD55 harboring TRAIL‐IETD‐Smac gene with broad antitumor effect. Hum Gene Ther. 2012;23:992‐1002. 22530834 10.1089/hum.2011.159 PMC3440030

[5] Zhang R , Zhang X , Ma B , et al. Enhanced antitumor effect of combining TRAIL and MnSOD mediated by CEA‐controlled oncolytic adenovirus in lung cancer. Cancer Gene Ther. 2016;23:168‐177. 27080225 10.1038/cgt.2016.11

[6] Ashkenazi A , Pai RC , Fong S , et al. Safety and antitumor activity of recombinant soluble Apo2 ligand. J Clin Invest. 1999;104:155‐162. 10411544 10.1172/JCI6926 PMC408479

[7] Pan G , O'Rourke K , Chinnaiyan AM , et al. The receptor for the cytotoxic ligand TRAIL. Science. 1997;276:111‐113. 9082980 10.1126/science.276.5309.111

[8] Ma H , Liu Y , Liu S , et al. Recombinant adeno‐associated virus‐mediated TRAIL gene therapy suppresses liver metastatic tumors. Int J Cancer. 2005;116:314‐321. 15800912 10.1002/ijc.20982

[9] Zhao L , Dong A , Gu J , et al. The antitumor activity of TRAIL and IL‐24 with replicating oncolytic adenovirus in colorectal cancer. Cancer Gene Ther. 2006;13:1011‐1022. 16799468 10.1038/sj.cgt.7700969

[10] Shi J , Zheng D , Liu Y , et al. Overexpression of soluble TRAIL induces apoptosis in human lung adenocarcinoma and inhibits growth of tumor xenografts in nude mice. Cancer Res. 2005;65:1687‐1692. 15753363 10.1158/0008-5472.CAN-04-2749

[11] Zhang Y , Gu J , Zhao L , et al. Complete elimination of colorectal tumor xenograft by combined manganese superoxide dismutase with tumor necrosis factor‐related apoptosis‐inducing ligand gene virotherapy. Cancer Res. 2006;66:4291‐4298. 16618754 10.1158/0008-5472.CAN-05-1834

[12] Huang F , Ma B , Wang Y , et al. Targeting gene‐virus‐mediated manganese superoxide dismutase effectively suppresses tumor growth in hepatocellular carcinoma in vitro and in vivo. Cancer Biother Radiopharm. 2014;29:403‐411. 25414976 10.1089/cbr.2014.1642

[13] Behrend L , Mohr A , Dick T , Zwacka RM . Manganese superoxide dismutase induces p53‐dependent senescence in colorectal cancer cells. Mol Cell Biol. 2005;25:7758‐7769. 16107721 10.1128/MCB.25.17.7758-7769.2005 PMC1190300

[14] Weydert C , Roling B , Liu J , et al. Suppression of the malignant phenotype in human pancreatic cancer cells by the overexpression of manganese superoxide dismutase. Mol Cancer Ther. 2003;2:361‐369. 12700280

[15] Gish RG , Porta C , Lazar L , et al. Phase III randomized controlled trial comparing the survival of patients with unresectable hepatocellular carcinoma treated with nolatrexed or doxorubicin. J Clin Oncol. 2007;25:3069‐3075. 17634485 10.1200/JCO.2006.08.4046

[16] Jurcut R , Wildiers H , Ganame J , D'Hooge J , Paridaens R , Voigt JU . Detection and monitoring of cardiotoxicity‐what does modern cardiology offer? Support Care Cancer. 2008;16:437‐445. 18197426 10.1007/s00520-007-0397-6

[17] Ribas A , Dummer R , Puzanov I , et al. Oncolytic Virotherapy Promotes Intratumoral T Cell Infiltration and Improves Anti‐PD‐1 Immunotherapy. Cell. 2017;170(1109–19):e10. 10.1016/j.cell.2017.08.027 PMC8034392 28886381

[18] Puzanov I , Milhem MM , Minor D , et al. Talimogene laherparepvec in combination with ipilimumab in previously untreated, unresectable stage IIIb‐IV melanoma. J Clin Oncol. 2016;34:2619‐2626. 27298410 10.1200/JCO.2016.67.1529 PMC7189507

[19] Bourgeois‐Daigneault MC , Roy DG , Aitken AS , et al. Neoadjuvant oncolytic virotherapy before surgery sensitizes triple‐negative breast cancer to immune checkpoint therapy. Sci Transl Med. 2018;10. 10.1126/scitranslmed.aao1641 29298865

[20] Selman M , Ou P , Rousso C , et al. Dimethyl fumarate potentiates oncolytic virotherapy through NF‐kappaB inhibition. Sci Transl Med. 2018;10. 10.1126/scitranslmed.aao1613 29367345

[21] Sakhawat A , Ma L , Muhammad T , Khan AA , Chen X , Huang Y . A tumor targeting oncolytic adenovirus can improve therapeutic outcomes in chemotherapy resistant metastatic human breast carcinoma. Sci Rep. 2019;9:7504. 31097752 10.1038/s41598-019-43668-8 PMC6522519

[22] Ying C , Xiao BD , Qin Y , et al. GOLPH2‐regulated oncolytic adenovirus, GD55, exerts strong killing effect on human prostate cancer stem‐like cells in vitro and in vivo. Acta Pharmacol Sin. 2018;39:405‐414. 28880012 10.1038/aps.2017.91 PMC5843840

[23] Zhang X , Meng S , Zhang R , et al. GP73‐regulated oncolytic adenoviruses possess potent killing effect on human liver cancer stem‐like cells. Oncotarget. 2016;7:29346‐29358. 27121064 10.18632/oncotarget.8830 PMC5045400

[24] Wang Y , Liu T , Huang P , et al. A novel Golgi protein (GOLPH2)‐regulated oncolytic adenovirus exhibits potent antitumor efficacy in hepatocellular carcinoma. Oncotarget. 2015;6:13564‐13578. 25980438 10.18632/oncotarget.3769 PMC4537034

[25] Wang L , Yao B , Li Q , et al. Gene therapy with recombinant adenovirus encoding endostatin encapsulated in cationic liposome in coxsackievirus and adenovirus receptor‐deficient colon carcinoma murine models. Hum Gene Ther. 2011;22:1061‐1069. 21615297 10.1089/hum.2011.014

[26] Nigatu AS , Vupputuri S , Flynn N , Neely BJ , Ramsey JD . Evaluation of cell‐penetrating peptide/adenovirus particles for transduction of CAR‐negative cells. J Pharm Sci. 2013;102:1981‐1993. 23592439 10.1002/jps.23556

[27] Zhang SN , Choi IK , Huang JH , Yoo JY , Choi KJ , Yun CO . Optimizing DC vaccination by combination with oncolytic adenovirus coexpressing IL‐12 and GM‐CSF. Mol Ther. 2011;19:1558‐1568. 21468000 10.1038/mt.2011.29 PMC3149171

[28] Yang Z , Zhang Q , Xu K , et al. Combined therapy with cytokine‐induced killer cells and oncolytic adenovirus expressing IL‐12 induce enhanced antitumor activity in liver tumor model. PLoS One. 2012;7:e44802. 23028626 10.1371/journal.pone.0044802 PMC3445563

[29] Chen K , Man K , Metselaar HJ , Janssen HL , Peppelenbosch MP , Pan Q . Rationale of personalized immunosuppressive medication for hepatocellular carcinoma patients after liver transplantation. Liver Transpl. 2014;20:261‐269. 24376158 10.1002/lt.23806

[30] Ma B , Wang Y , Zhou X , et al. Synergistic suppression effect on tumor growth of hepatocellular carcinoma by combining oncolytic adenovirus carrying XAF1 with cisplatin. J Cancer Res Clin Oncol. 2015;141:419‐429. 25240826 10.1007/s00432-014-1835-8 PMC11824151

[31] Xiao B , Qin Y , Ying C , et al. Combination of oncolytic adenovirus and luteolin exerts synergistic antitumor effects in colorectal cancer cells and a mouse model. Mol Med Rep. 2017;16:9375‐9382. 29039580 10.3892/mmr.2017.7784 PMC5779991

[32] Ali S , Tahir M , Khan AA , Chen XC , Ling M , Huang Y . Cisplatin synergistically enhances antitumor potency of conditionally replicating adenovirus via p53 dependent or independent pathways in human lung carcinoma. Int J Mol Sci. 2019;20:1125. 10.3390/ijms20051125 PMC6429304 30841620

[33] Chen K , Sheng J , Ma B , et al. Suppression of hepatocellular carcinoma by mycophenolic acid in experimental models and in patients. Transplantation. 2019;103:929‐937. 30747839 10.1097/TP.0000000000002647

[34] Ding X , Zhang Y , Yang H , et al. Long non‐coding RNAs may serve as biomarkers in breast cancer combined with primary lung cancer. Oncotarget. 2017;8:58210‐58221. 28938549 10.18632/oncotarget.17356 PMC5601645

[35] Ding X , Zhu L , Ji T , et al. Long intergenic non‐coding RNAs (LincRNAs) identified by RNA‐seq in breast cancer. PLoS One. 2014;9:e103270. 25084155 10.1371/journal.pone.0103270 PMC4118859

[36] Yang F , Yi F , Zheng Z , et al. Characterization of a carcinogenesis‐associated long non‐coding RNA. RNA Biol. 2012;9:110‐116. 22258142 10.4161/rna.9.1.18332

[37] Li Y , Jiang Z , Xu L , Yao H , Guo J , Ding X . Stability analysis of liver cancer‐related microRNAs. Acta Biochim Biophys Sin (Shanghai). 2011;43:69‐78. 21173058 10.1093/abbs/gmq114

[38] Wang F , Zheng Z , Guo J , Ding X . Correlation and quantitation of microRNA aberrant expression in tissues and sera from patients with breast tumor. Gynecol Oncol. 2010;119:586‐593. 20801493 10.1016/j.ygyno.2010.07.021

