# Supplementary figures and images for: Doxorubicin hydrochloride enhanced antitumour effect of CEA‐regulated oncolytic virotherapy in live cancer cells and a mouse model
Source: J Cell Mol Med. 2020 Oct 14;24(22):13431–9. doi: 10.1111/jcmm.15966 (PMC7701578; doi:10.1111/jcmm.15966)

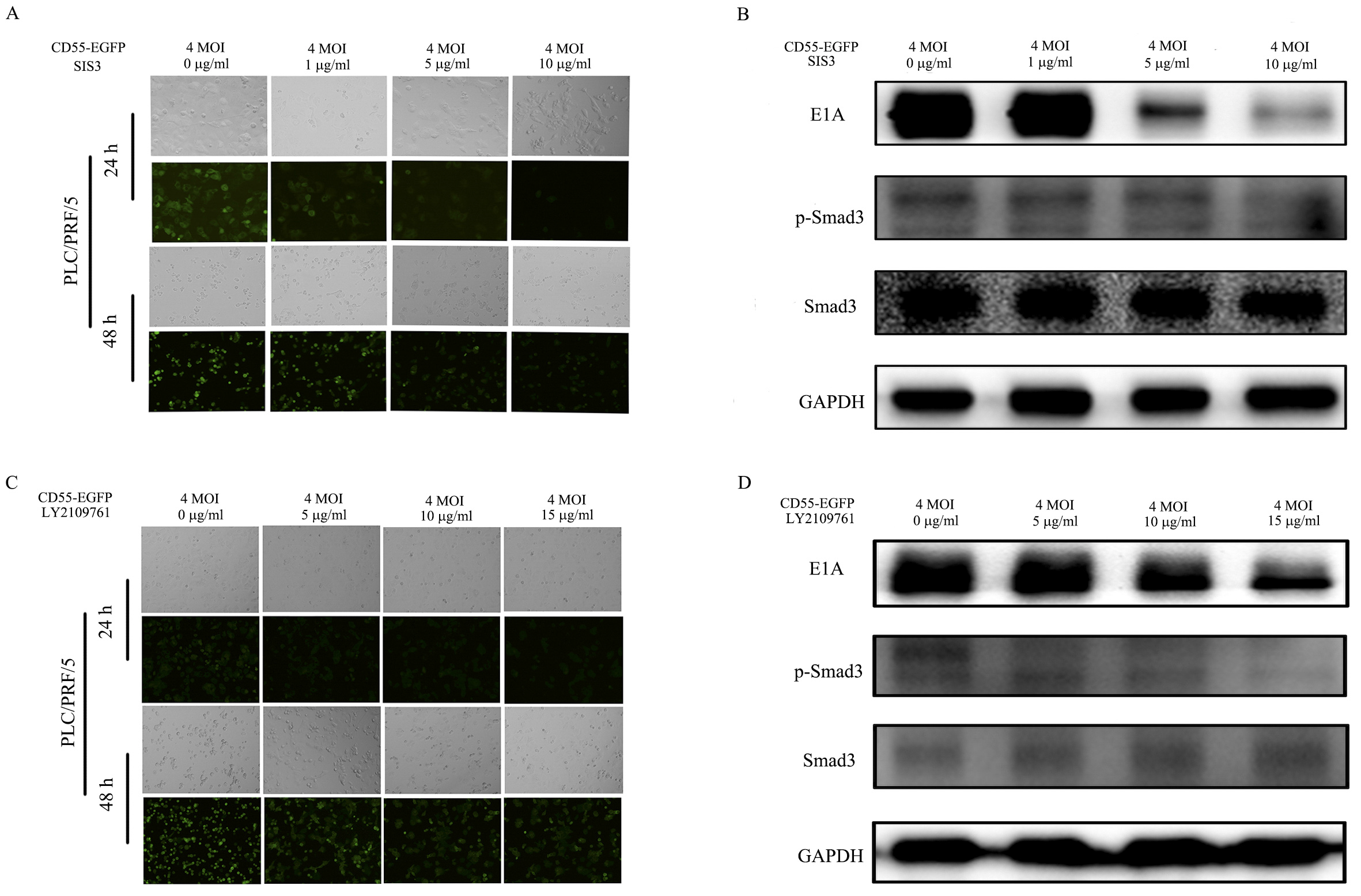

Supplement: Supplementary file 1 — Fig S1 [file JCMM-24-13431-s001.tif]

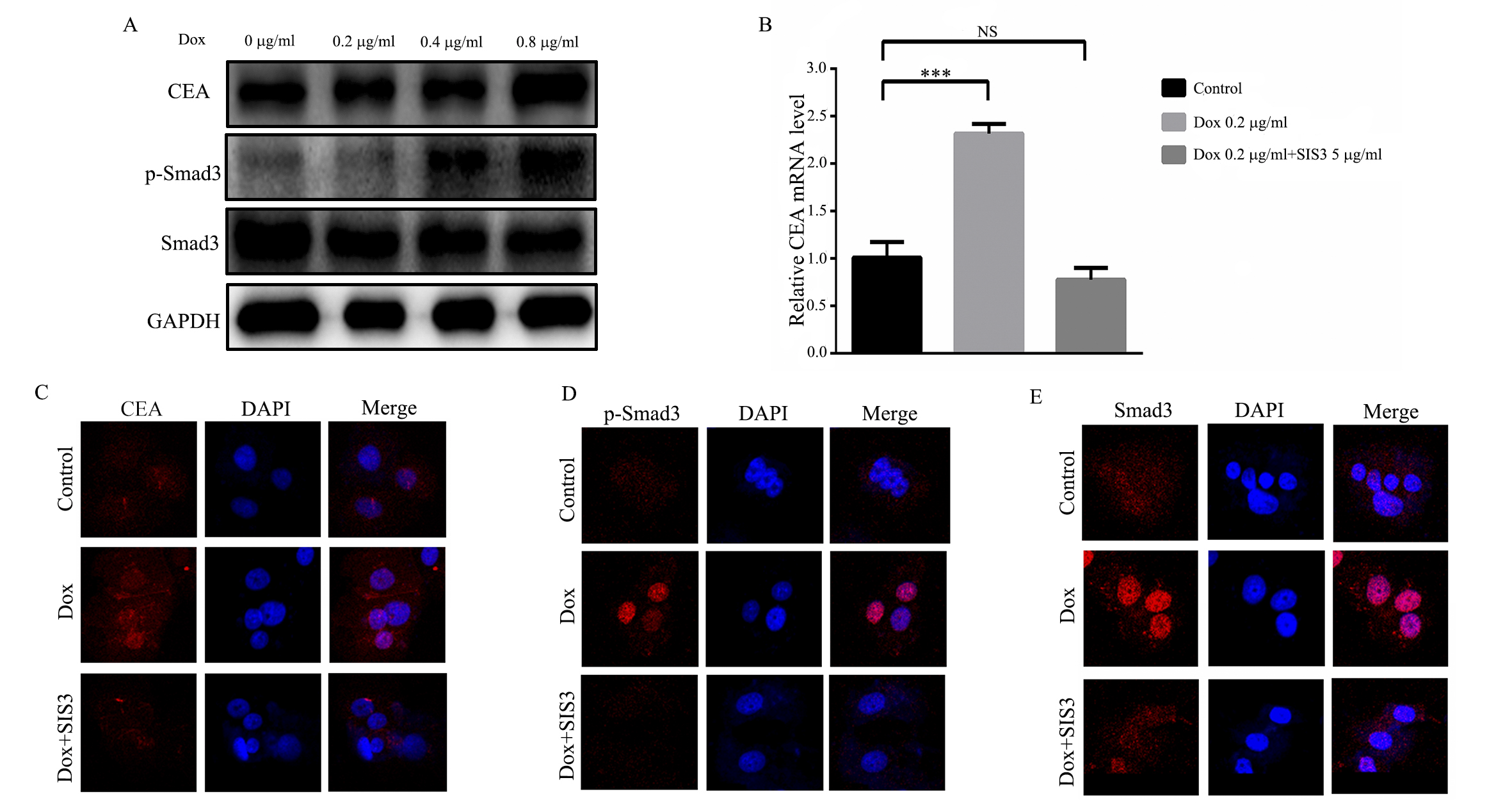

Supplement: Supplementary file 2 — Fig S2 [file JCMM-24-13431-s002.tif]

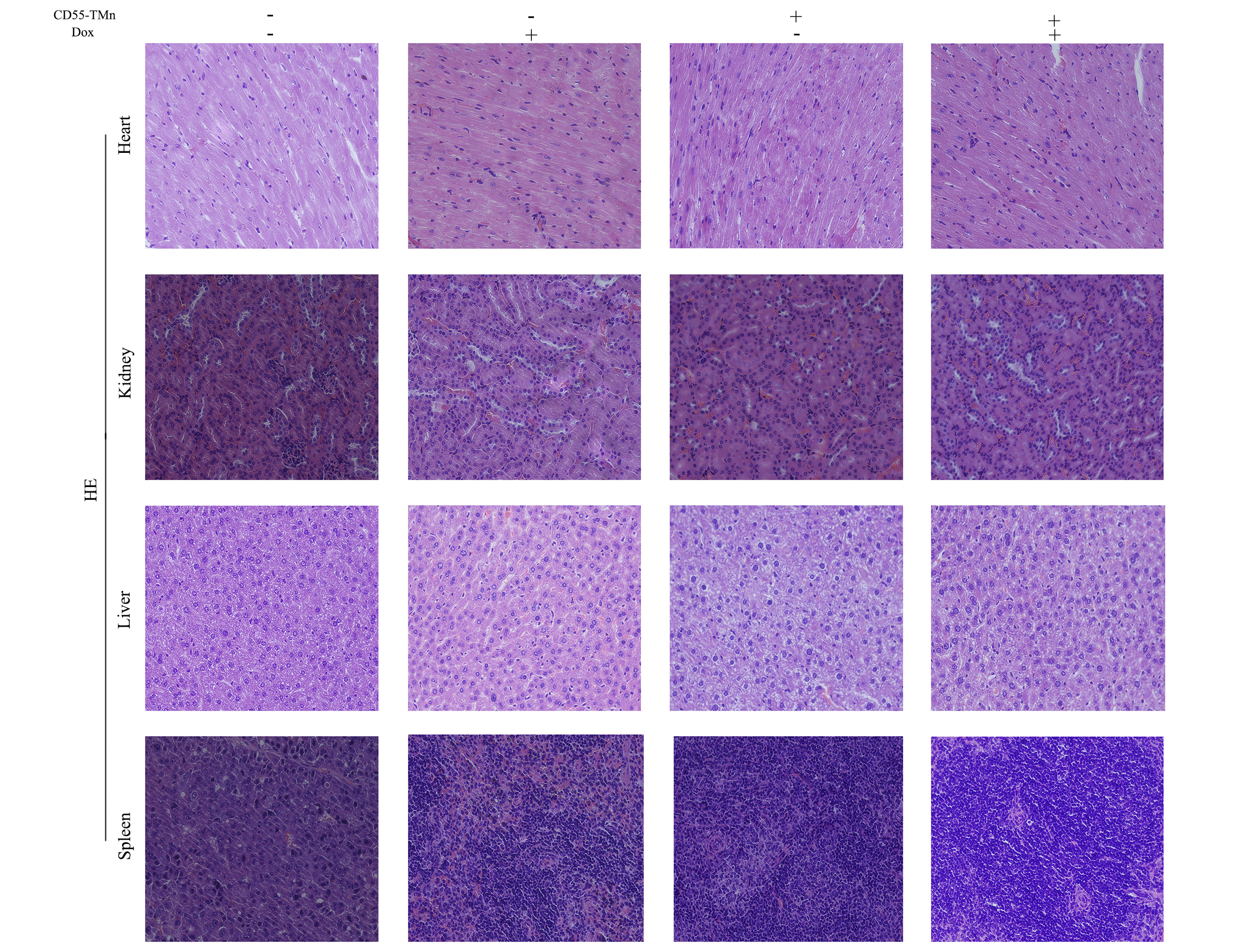

Supplement: Supplementary file 3 — Fig S3 [file JCMM-24-13431-s003.tif]
